# Systematic evaluation of the association between a missense variant in *XRCC3* gene splicing site and the pathogenesis of ovarian cancer

**DOI:** 10.1042/BSR20230462

**Published:** 2023-06-13

**Authors:** Qiulian Liang, Gongchen Huang, Ping Zhong, Dengting Deng, Lin Yang, Xiangyuan Yu

**Affiliations:** 1 The Guangxi Key Laboratory of Environmental Exposomics and Entire Lifecycle Health, Guilin Medical University, Guilin 541199, China; 2 Department of Obstetrics and Gynecology, The Second Affiliated Hospital of Guilin Medical University, Guilin 541199, China; 3 Department of Gynecology, Guilin Hospital of traditional Chinese Medicine, Guilin 541002, Guangxi of China

**Keywords:** Association, DNA damage repair, functional analysis, ovarian cancer, variant

## Abstract

The effects and underlying mechanism of *XRCC3* rs861539 on the risk of ovarian cancer (OC) are still unclear. Therefore, a meta-analysis of 10 studies containing 6,375 OC cases and 10,204 controls was performed for this topic. Compared with GG genotype, GA + AA genotypes could significantly decrease the OC risk, odds ratios (ORs) and their corresponding 95% confidence intervals (CIs) were 0.89 (0.83-0.95) and *P*=0.001, and 0.88 (0.82–0.95) and *P*=0.001 under the dominant and heterozygous genetic models. Compared with G allele, rs861539 A could significantly reduce the OC risk, OR and its corresponding 95% CI was 0.94 (0.89–0.98) and *P*=0.007. By subgroup analysis in ethnicity, protective effects on OC risk in Caucasians were observed (the dominant model: OR = 0.88, 95% CI = 0.82–0.94, *P*<0.001; the heterozygous model: OR = 0.87, 95% CI = 0.81–0.94, *P*<0.001; the allelic model: OR = 0.93, 95% CI = 0.88–0.97, *P*=0.003; the homozygous model: OR = 0.89, 95% CI = 0.80–0.98, *P*=0.024). The authenticity of positive association findings was further confirmed by trial sequential analysis (TSA) and false-positive report probability (FPRP) analysis. The subsequent functional analysis revealed that rs861539 could regulate the post-transcriptional expression of *XRCC3* by changing the activity of putative splice sites and types of splicing factors. rs861539 also may act as an expression Quantitative Trait Loci (eQTL) affecting the expression of genes such as *XRCC3*, *MARK3*, *APOPT1*, etc., and has an impact on the structure of XRCC3.

## Introduction

Worldwide, ovarian cancer (OC) is one of the most common gynecologic cancers, and has the highest mortality rate between them and occupy the third place in mortality, only after cervical and uterine cancer [1]. At present, the global morbidity of OC is approximately 3.4%, and is on the rise [2]. It is now clear that the occurrence of OC can be caused by a variety of physiological and environmental high-risk factors such as sustained ovulation, industrial physical and chemical pollutants, etc. However, under the same level of environmental exposure, individuals have different susceptibility to OC. This suggests that the difference of genetic background may also play a key role in the pathogenesis of OC [3].

Human genomic DNA is often damaged by internal and exogenous factors, such as metabolic free radicals, viruses, ultraviolet radiation, ionizing radiation, etc. If the damaged DNA cannot be repaired effectively in time or erroneously, it may affect the specific functions of gene execution and increase the risk of cell canceration [4]. Single-nucleotide polymorphism (SNP) is the most common genetic variation in humans, which can regulate gene expression or change the function of gene products to determine individual susceptibility to disease. The X-ray cross-complementing group 3 (XRCC3) plays a key role in repairing the DNA double-strand breaks and maintaining the functional integrity of the genome. Studies showed that the SNPs of *XRCC3* gene may affect its DNA damage repair efficiency and be associated with the pathogenesis of tumors [5–7].

Single nucleotide variant rs861539 G>A is located at the 241st amino acid in the coding region of *XRCC3* gene, which can cause the coding amino acid changed from Threonine to Methionine (Thr241Met), and is defined as a missense mutation. In addition, rs861539 is also a SNP of a splicing site of *XRCC3* gene [8]. This suggests that rs861539 may play an important regulatory role in the post-transcriptional expression of *XRCC3* gene and the functional execution of XRCC3 protein products. Growing evidence suggests that rs861539 changes the function of XRCC3 and is involved in DNA damage and subsequent susceptibility to carcinogens.

Currently, a series of studies have investigated the relationship between the rs861539 and OC pathogenesis, but the conclusions are still contradictory [9–18]. So far, the relationship between rs861539 and the pathogenesis of OC, the strength of the relationship and the underlying functional mechanism are not clear. In view of this, based on the principles and methods of evidence-based medicine, this study objectively evaluated and clarified the impact of *XRCC3* rs861539 G>A on the incidence of ovarian cancer, identified the potential mechanism, and provided scientific basis for the prevention of OC.

## Materials and methods

### Meta-analysis

#### Literature search strategy

Joint search of China national knowledge infrastructure (CNKI), Wanfang Medical Network, Web of Science and NCBI PubMed database, with ‘*XRCC3*’, ‘DNA repair gene’, ‘polymorphism’, ‘ovarian cancer’, ‘risk’ as the search subject words or keywords, to screen the published studies on the relationship between the *XRCC3* gene rs861539 and the pathogenesis of OC. The deadline is April 10, 2023. In addition, manually retrieve the references to be included in the studies to obtain the target literature.

#### Inclusion and exclusion criteria

The studies included in the meta-analysis should meet the following criteria: (1) published case–control studies on the relationship between *XRCC3* rs861539 variant and OC risk, (2) definite clinical and pathological diagnosis in the patients of OC, (3) comparable non-tumor subjects in control group, and (4) providing full genotype or allele frequency data in included studies. The exclusion criteria were as follows: (1) animal and cell experiments, (2) the studies being irrelevant to the relationship between *XRCC3* rs861539 and OC genetic predisposition, (3) unavailable genotyping data, and (4) abstract, case report, review, systematic review or meta-analysis.

#### Quality appraisal and data extraction

The quality of the included studies was assessed by two investigators (QL Liang and GC Huang) by using the Newcastle–Ottawa scale (NOS). Totally, eight items were used to evaluate the selection, comparability and exposure of the study population. Specifically, if one item is met, 1 point can be obtained (except for the item of comparability containing two points), with 0–9 points. Among them, 0–3 points are rated as low quality, 4–6 points as medium quality and 7–9 points as high quality documents.

The two investigators independently extracted necessary data from the eligible studies and then crosschecked to resolve the disagreements. The information of first author, paper publication year, country, ethnicity, age (years old), genotype frequency of cases and controls, number of case and control groups in the included studies was extracted.

### Data processing

#### Meta-analysis

The odds ratios (ORs) and corresponding 95% confidence intervals (CIs) were used to assess the strength of association between rs861539 and the OC risk. The heterogeneity across the studies was assessed by the *Q*-test, and was considered significant when a *P*-value less than 0.1 [19]. A fixed-effect model was used to calculate the pooled OR if the heterogeneity was not significant; otherwise, the random-effect model was adopted [20]. The subgroup analysis in ethnicity was also performed to detect whether Caucasian populations and Asian populations have different susceptibility to OC. Meanwhile, publication bias was tested by Begg’s method with funnel plot to identify significant publication bias [21], and the sensitivity analyses were also done to assess the influence of individual study on the pooled ORs. All data processing was undertaken by using the Stata software, version 12.0 (Stata Corp LP, College Station, TX, U.S.A.).

#### False-positive report probability (FPRP) analysis

In addition, the false-positive report probability (FPRP) analysis was carried out to evaluate the robustness of positive associations found in the pooled analyses. The cut-off value of 0.2 and prior probability of 0.1 was set to detect the efficacy for OR of 1.2 or 1/1.2 that is most likely. True associations would be considered when FPRP value was <0.2 [22].

### Trial sequential analysis

Random errors may increase considerably when new emerging researches were continuously included to calculate the significance, causing a false conclusion in the meta-analysis [23]. Therefore, a trial sequential analysis (TSA) was performed to decrease the random errors in present study. In parameter settings, the Type I error (α = 5%), the Type II error (β = 20%), the relative risk reduction (RRR = 20%) and two-sided graph plots were set, respectively. TSA can help to calculate the required information size (RIS) and adjust *P*-value with trial sequential monitoring boundary. When the TSA monitoring boundary crosses with cumulative Z-curve before the RIS is reached, it suggests that significant evidence is confirmed and further trials are not necessary [24]. The related data processing was undertaken by using the TSA software (version 0.9.5.10 beta).

### Biological function analysis

Given the fact that the rs861539 is a missense variant and located in a potential splice site of the *XRCC3* gene, the SNPinfo Web Server (https://manticore.niehs.nih.gov/snpinfo/guide.html) [8], Alternative Splice Site Predictor (ASSP) tool (http://wangcomputing.com/assp/index.html) [25] and VarNote-REG (http://www.mulinlab.org/varnote/application.html#REG) [26] online tools were adopted to analyze the effect of rs861539 G>A on expression regulation of *XRCC3* gene. Meanwhile, Polyphen-2 (http://genetics.bwh.harvard.edu/pph2/) [27] was taken to predict the possible classification of effect on protein′s structure and function according to different sequence and structural features of the amino acid substitutions positions. The protein ID of XRCC3 was obtained from Uniport database, and the protein structure with wild-type to mutant amino acid was analyzed by Pymol 1.4.1 [28] (The PyMOL Molecular Graphics System), which would help to visualize the changes in the amino acid. The pdb filetext was downloaded from Protein Data Bank (PDB).

## Results

### Meta-analysis

#### Study characteristics

**Figure 1:**
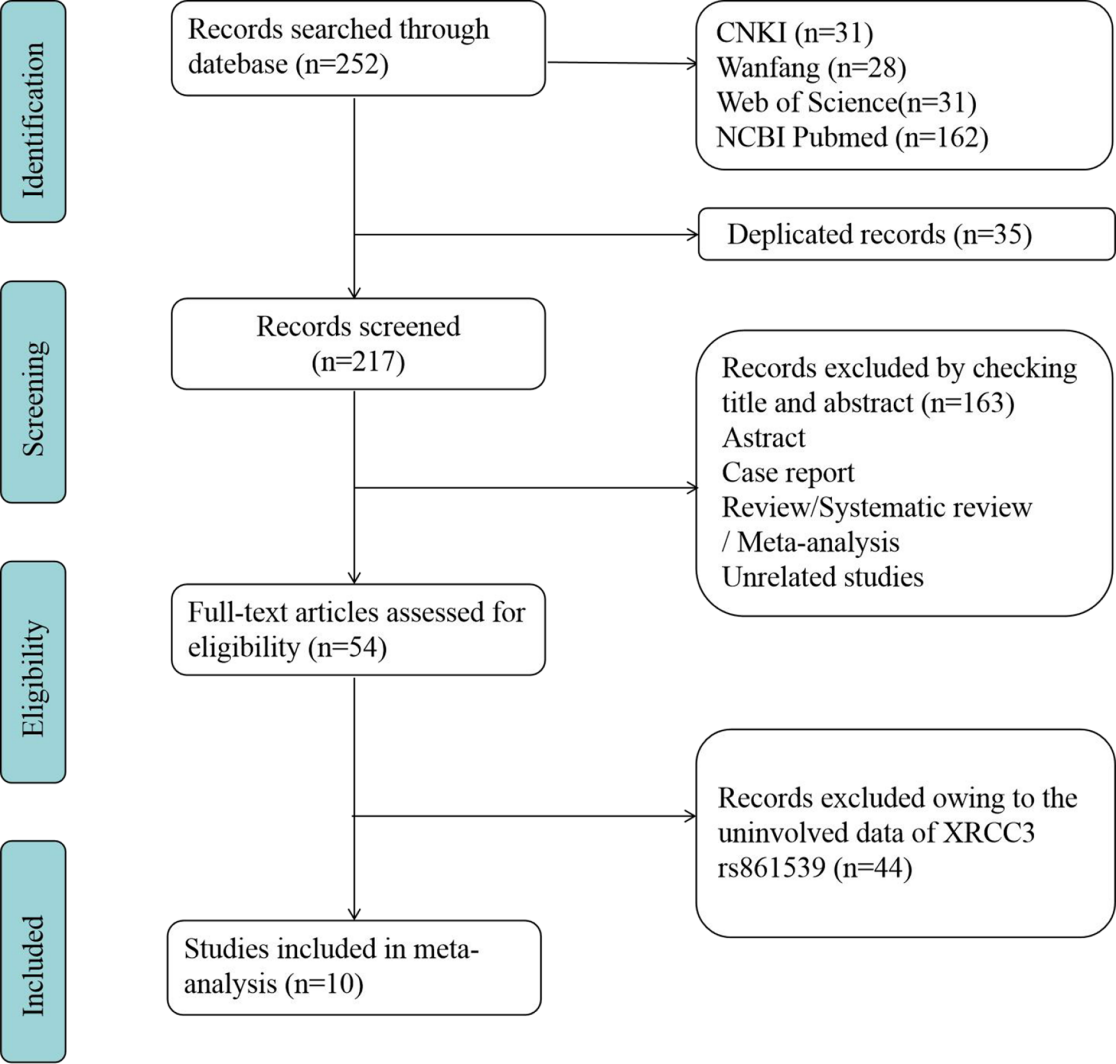
The flowchart of literature screening

After searching of China national knowledge internet (CNKI), Wanfang Medical Network, Web of Science and NCBI PubMed databases, a total of 252 studies on the relationship between *XRCC3* gene and OC were preliminarily retrieved. Due to 35 duplications, 217 studies were continuously screened. The literature was retrieved strictly followed the inclusion and exclusion criteria. First of all, 163 papers such as abstracts, case reports, reviews, systematic reviews or meta-analyses and unrelated studies were excluded by checking title and abstract. And then, by reading the original text in detail, 44 studies did not involve the necessary genotyping data of rs861539 variant were excluded. Finally, 10 eligible studies involving 14 research data and 16,579 subjects (6,375 cases and 10,204 controls) were recruited in present meta-analysis. See Figure 1 for the details.

The quality of studies included in this meta-analysis was assessed by utilizing the NOS. After quality evaluation, two study data were considered as low quality, four data as medium quality and eight data as high quality. The main characteristics of included studies are shown in Table 1.

**Table 1:** General characteristics of studies included in the meta-analysis

Study	Year	Country	Ethnicity	Genotype (Case)				Genotype (Control)				Age (case/control)	*P*_HWE_ of control	Quality score
				GG	GA	AA	All	GG	GA	AA	All			
Gowtham Kumar, G [9]	2021	India	Asian	189	192	67	448	362	460	130	952	20–80/20–80	0.131	6
Mackawy, AMH [10]	2019	Egypt	African	297	347	105	794	318	404	108	830	45.22 ± 5.4/47.7 ± 6.96	0.094	6
Smolarz, B [11]	2019	Poland	Caucasian	125	114	31	270	130	174	40	344	41.2(37–79)/ 43.1(35–77)	0.118	8
Michalska, MM [12]	2016	Poland	Caucasian	144	168	49	361	358	394	139	891	53.20 ± 9.11/50.42 ± 17.22	0.892	9
Gao, ZB [13]	2015	China	Asian	130	121	39	290	728	827	229	1784	54 ± 12/54 ± 12	0.52	3
Monteiro, MS [14]	2014	Brazil	Miscegenation	207	223	74	504	370	471	131	972	41.0 ± 14.5/53.9 ± 15.0	0.368	3
Quaye, L [15]	2009	Denmark, UK, USA	Caucasian	291	339	101	731	288	351	108	747	23–74/23–80	0.696	9
Beesley, J [16]^*^	2007	Australia	Caucasian	180	340	180	700	150	350	200	700	57/44	0.326	7
Beesley, J [16]^†^	2007	Australia	Caucasian	147	307	146	600	117	317	166	600	58/57	0.95	9
Webb, PM [17]	2005	Australia	Caucasian	62	6	2	70	60	10	0	70	18–95/30–90	0.398	7
Auranen, A [18]^*^	2005	UK	Caucasian	545	612	175	1332	784	958	282	2024	<70/45–74	0.248	9
Auranen, A [18]^†^	2005	USA	Caucasian	32	33	5	70	32	33	5	70	20–64	0.111	6
Auranen, A [18]^‡^	2005	Denmark	Caucasian	136	49	15	200	140	51	9	200	35–79/35–79	0.079	7
Auranen, A [18]^§^	2005	UK	Caucasian	21	17	12	50	14	4	2	20	<70	0.806	5

Note: HWE, Hardy–Weinberg equilibrium.

*,†,‡,§:Research data 1,2,3,4 of a literature respectively.

#### Systematic assessment

The Chi-square Q test showed that there was no significant heterogeneity in the included data under the five genetic models (dominant genetic model [GA + AA vs. GG: *I^2^* = 0.30%, *P*_heterogeneity_ = 0.445], the recessive model [AA vs. GG + GA: *I^2^* = 0.00%, *P*_heterogeneity_ = 0.734], the heterozygous model [GA vs. GG: *I^2^* = 0.00%, *P*_heterogeneity_ = 0.575], the homozygous model [AA vs. GG: *I^2^* = 0.00%, *P*_heterogeneity_ = 0.520] and the allelic model [A vs. G: *I^2^* = 0.00%, *P*_heterogeneity_ = 0.503]). Thus, the subsequent meta-analysis was performed using a fixed-effects model.

**Figure 2:**
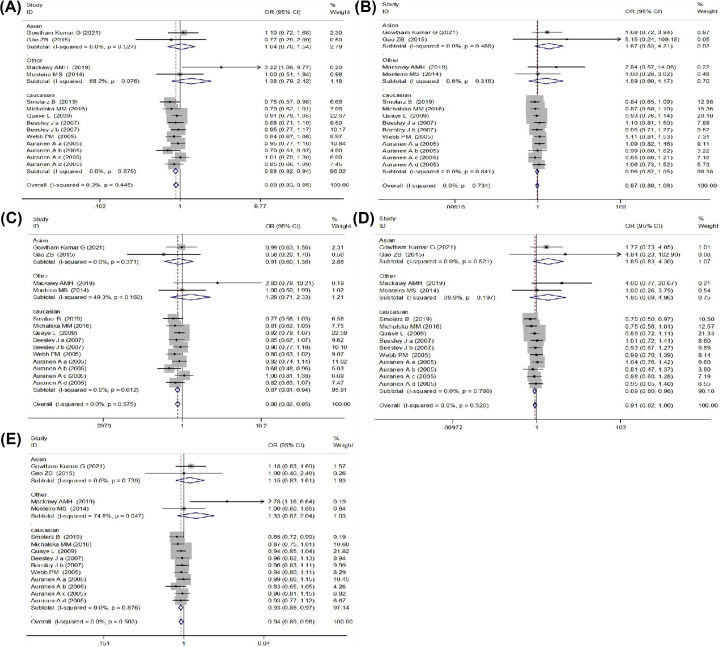
The forest plot depicting the association between rs861539 and OC risk (**A**) Dominant model (GA + AA vs. GG); (**B**) Recessive model (AA vs. GA+GG); (**C**) Heterozygous model (GA vs. GG); (**D**) Homozygous model (AA vs. GG); (**E**) Allelic model (A vs. G).

The results of meta-analysis showed that rs861539 was significantly associated with the genetic susceptibility of OC. Comparing with the GG genotype, GA + AA genotypes could significantly reduce the risk of OC, the ORs and their 95% CIs were 0.89 (0.83–0.95), *P=*0.001 in the dominant genetic model, 0.88 (0.82–0.95), *P=*0.001 in the heterozygous genetic model. Under the allelic model, compared with the G allele, the A allele could significantly reduce the OC risk, the OR and 95% CI was 0.94 (0.89–0.98), *P*=0.007. However, no significant association between rs861539 and OC risk was found under the recessive genetic model and the homozygous genetic model. As shown in Figure 2 and Table 2.

**Table 2:** The significant associations analysis between *XRCC3* rs861539 and the risk of OC

Analysis model	Sample size (Case/Control)	OR(95%CI)	*P*	Heterogeneity test	
				*I* ^2^	*P*_heterogeneity_
**Overall analysis**					
Dominant model (GA+AA vs. GG)	6375/10204	0.89 (0.83-0.95)	0.001	0.30%	0.445
Heterozygous model (GA vs. GG)	5374/8655	0.88 (0.82-0.95)	0.001	0.00%	0.575
Allelic model (A vs. G)	12750/20408	0.94 (0.89-0.98)	0.007	0.00%	0.503
**Subgroup analysis (Caucasian)**					
Dominant model (GA+AA vs. GG)	6375/10204	0.88 (0.82-0.94)	<0.001	0.00%	0.675
Homozygous model (AA vs. GG)	3507/5400	0.89 (0.80-0.98)	0.024	0.00%	0.786
Heterozygous model (GA vs. GG)	5374/8655	0.87 (0.81-0.94)	<0.001	0.00%	0.612
Allelic model (A vs. G)	12750/20408	0.93 (0.88-0.97)	0.003	0.00%	0.876

The subgroup analysis in ethnicity suggested a protective effect of rs861539 on OC risk in Caucasian populations under the dominant genetic model (OR = 0.88, 95% CI: 0.82–0.94, *P*<0.001), the homozygous model (OR = 0.89, 95% CI: 0.80–0.98, *P*=0.024), the heterozygous model (OR = 0.87, 95% CI: 0.81–0.94, *P*<0.001) and the allelic model (OR = 0.93, 95% CI: 0.88–0.97, *P*=0.003) except for the recessive model. However, there was no significant association observed in the Asian populations. As demonstrated in Figure 2.

#### Publication bias

**Figure 3:**
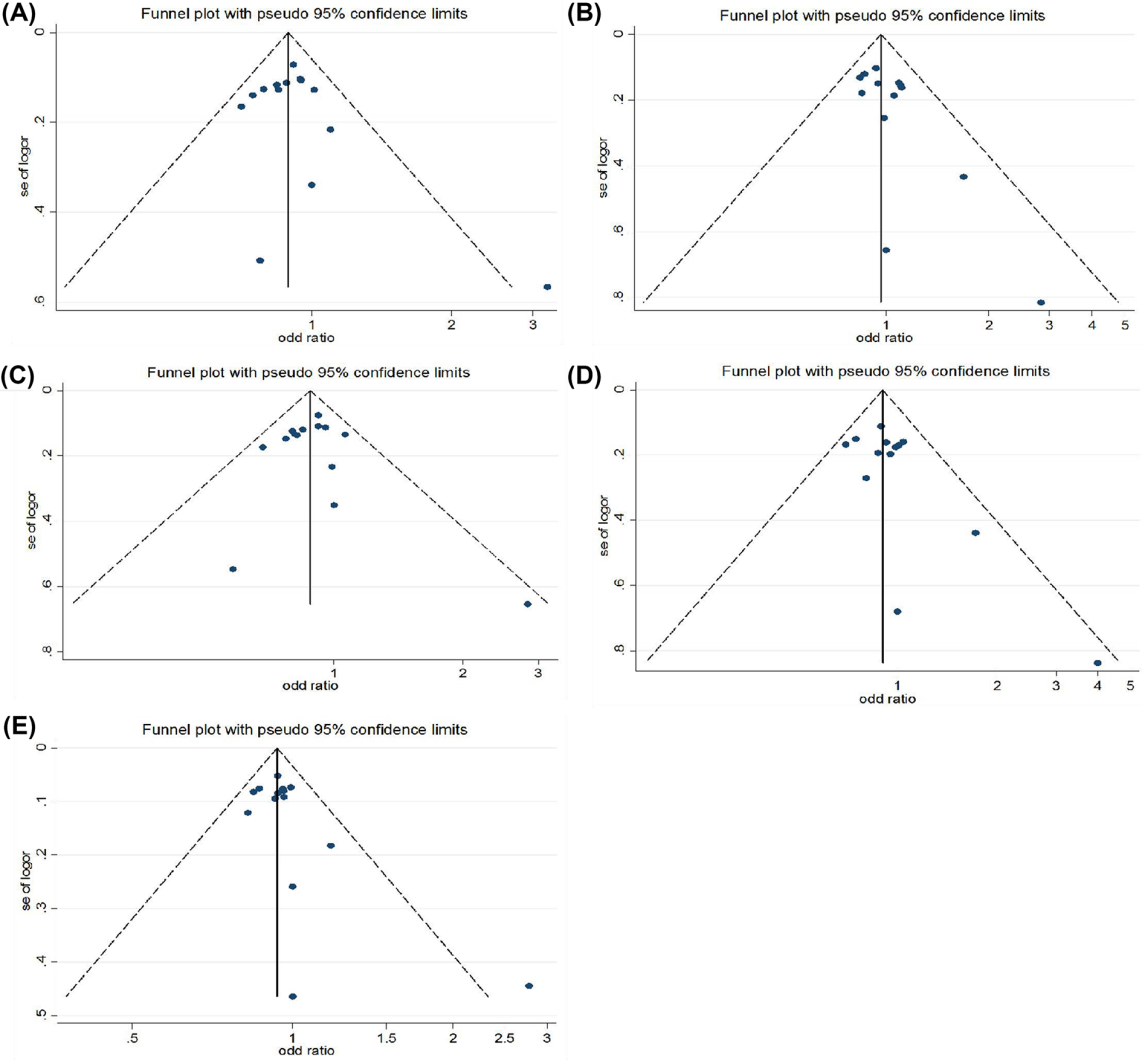
Funnel plots to detect publication bias (**A**) Dominant model (GA + AA vs. GG); (**B**) Recessive model (AA vs. GA+GG); (**C**) Heterozygous model (GA vs. GG); (**D**) Homozygous model (AA vs. GG); (**E**) Allelic model (A vs. G)

The evaluation of publication bias by Begg's test showed that the included studies were generally symmetrical distributed on both sides of the symmetry axis. It suggests that there may not be significant publication bias in the included literature in present study. As shown in Figure 3.

#### Sensitivity analysis

In this study, the sensitivity analysis was conducted by eliminating one study at a time to detect the influence of a single study data on the overall pooled effect. The results showed that no single study significantly changed the pooled OR and its corresponding 95% CI, indicating that the results of this meta-analysis were robust and the study conclusions were relatively reliable (data was not shown).

#### FPRP analysis

To verify the statistically significant associations, we performed the FPRP analysis with the presetting threshold of 0.2 and prior probability of 0.1. All significant associations found in the meta-analysis were considered to be true, and FPRP values for effects of rs861539 on OC risk in the dominant model, heterozygous model and allelic model were 0.009, 0.010 and 0.059, respectively. Moreover, it was found that the FPRP values were 0.001, 0.189, 0.001 and 0.026 for the effects of rs861539 on Caucasians OC risk in the subgroup analysis stratified by ethnicity under the dominant, homozygous, heterozygous and allelic models, respectively. The surprising findings indicated that the rs861539 leading a credibly decreased OC risk among Caucasian populations. As shown in Table 3.

**Table 3:** FPRP analysis for the significant associations of the rs861539 and OC risk

Genotype	OR (95% CI)	*P*_Value_	Statistical power	Prior probability					
				0.25	0.1	0.01	0.001	0.0001	0.00001
**Overall analysis**									
Dominant model (GA+AA vs. GG)	0.89 (0.83–0.95)	0.001	0.986	0.003	0.009	0.091	0.503	0.910	0.990
Heterozygous model (GA vs. GG)	0.88 (0.82–0.95)	0.001	0.916	0.003	0.010	0.098	0.522	0.916	0.991
Allelic model (A vs. G)	0.94 (0.89–0.98)	0.007	1.000	0.021	0.059	0.409	0.875	0.986	0.999
**Subgroup analysis** (Caucasian)									
Dominant model (GA+AA vs. GG)	0.88 (0.82–0.94)	<0.001	0.937	0.000	0.001	0.010	0.096	0.516	0.914
Homozygous model (AA vs. GG)	0.89 (0.80–0.98)	0.024	0.927	0.072	0.189	0.719	0.963	0.996	1.000
Heterozygous model (GA vs. GG)	0.87 (0.81–0.94)	<0.001	0.766	0.000	0.001	0.013	0.115	0.566	0.929
Allelic model (A vs. G)	0.93 (0.88–0.97)	0.003	1.000	0.009	0.026	0.229	0.750	0.968	0.997

#### Trial Sequential Analysis (TSA)

**Figure 4:**
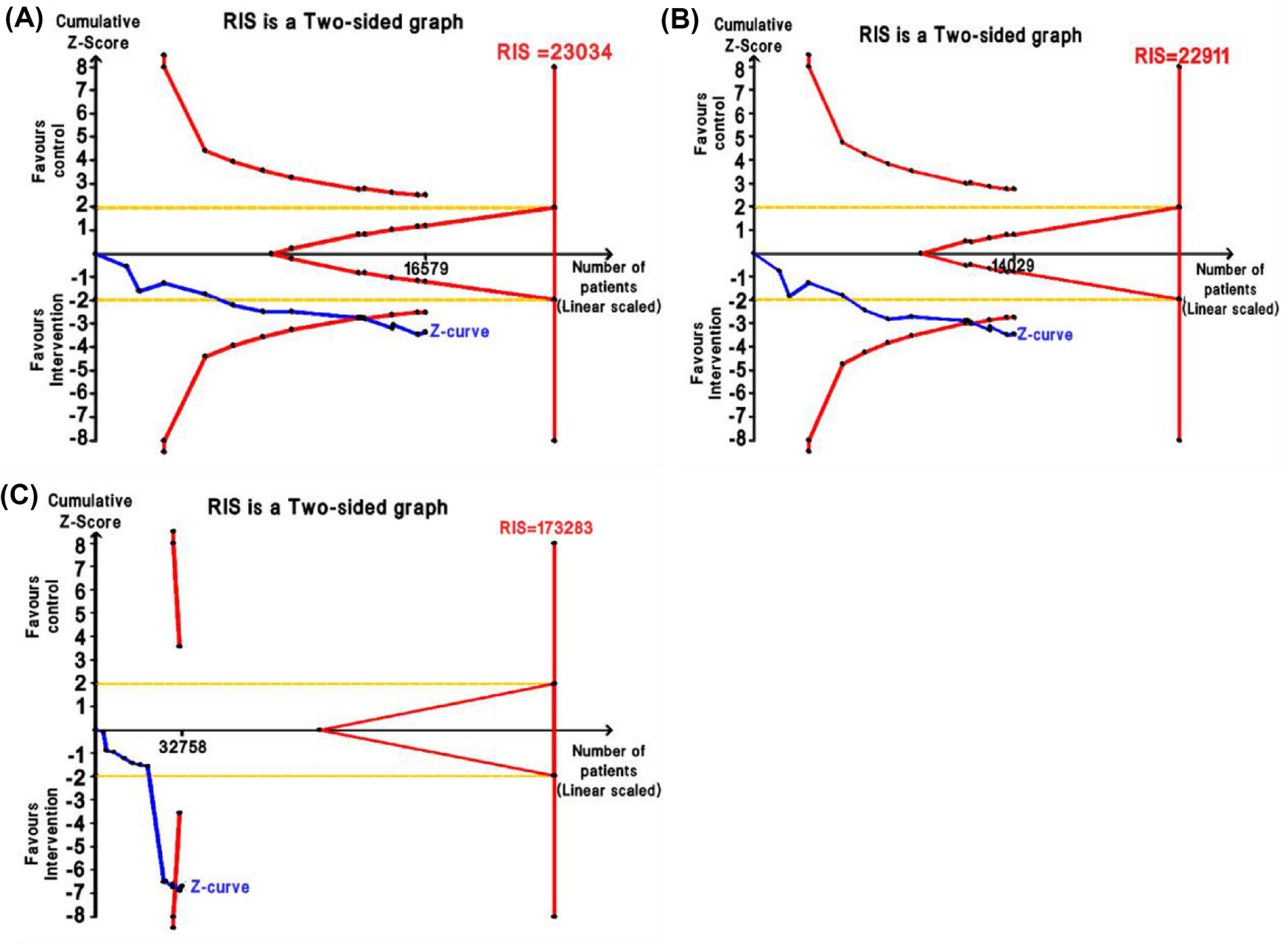
TSA analysis in the significant associations of rs861539 and OC risk (**A**) Dominant model (GA + AA vs. GG); (**B**) Heterozygous model (GA vs. GG); (**C**) Allelic model (A vs. G); Blue line: the cumulative Z-score; Red line: trial sequential monitoring boundaries; Yellow line: Conventional

The results of Trial Sequential Analysis (TSA) support the conclusions drawn from the meta-analysis. As depicted in Figure 4, although the cumulative Z-score line did not reach RIS, it crossed the traditional boundary and the TSA monitoring boundary, indicating that accumulative evidence was adequate to the significant associations of rs861539 and OC risk in the dominant model, the heterozygous model and the allelic model, and no further trials were required to verify these conclusions.

#### Functional analysis of rs861539 G>A

**Figure 5:**
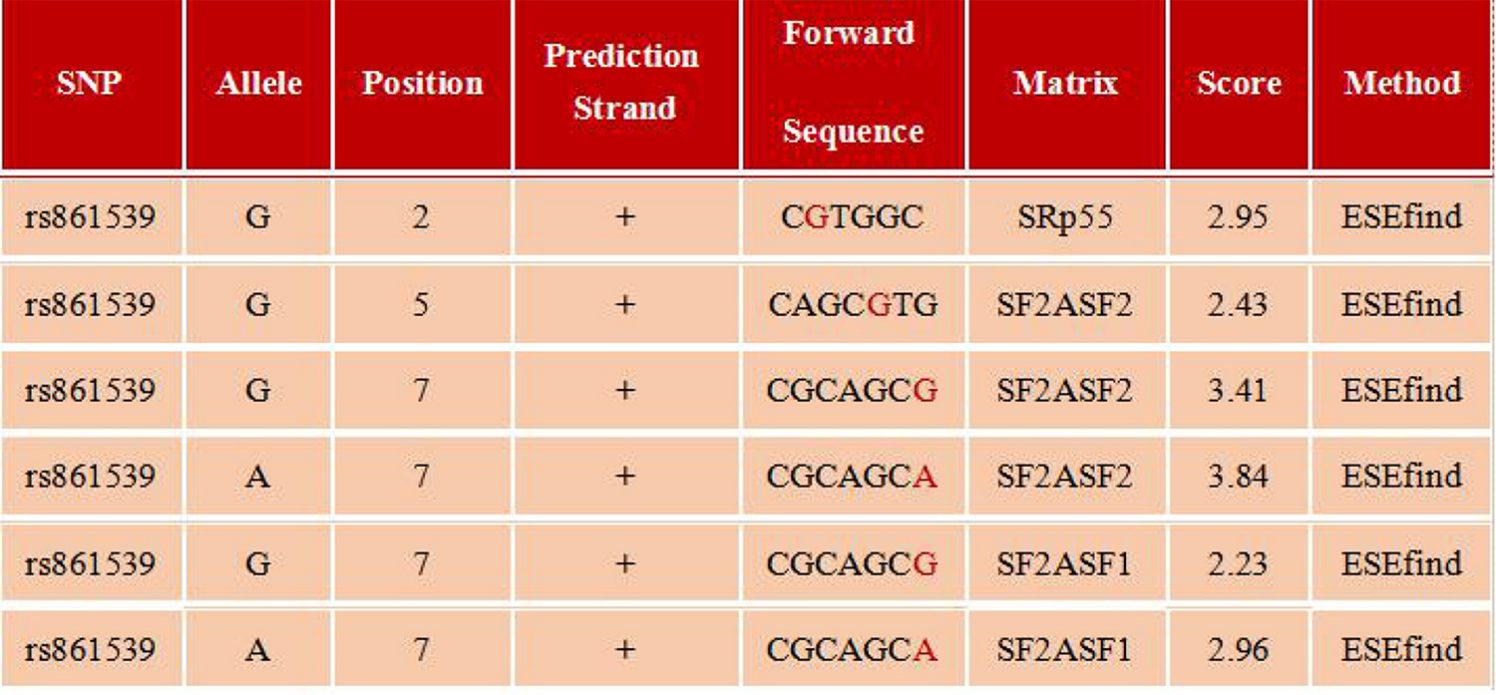
Predicted the function of rs861539 on XRCC3 post-transcriptional splicing sites by SNPinfo Web Serve

**Figure 6:**
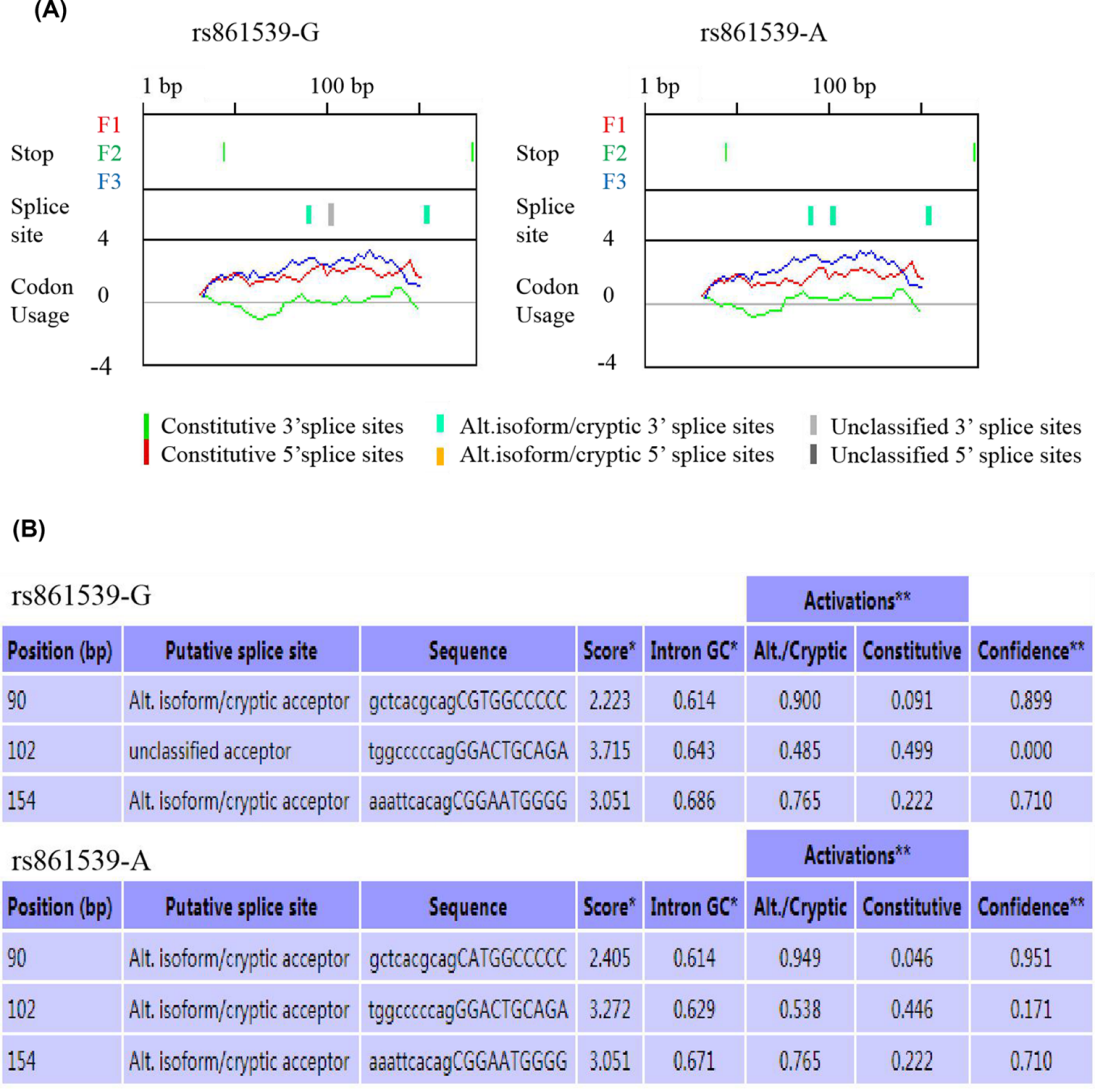
Analysis of splicing regulation function of *XRCC3* rs861539 by ASSP (Alternative Splice Site Predictor) (**A**) Graphical view represented the change of putative splice sites. (**B**) Scores of the preprocessing models reflecting splice site.

Since the missense mutation rs861539 of *XRCC3* gene splicing site is significantly associated with OC susceptibility, we evaluated the effect of this variant on the *XRCC3* gene post-transcriptional splicing and structure or function of the coding protein of XRCC3. The analysis of Exonic Splicing Enhancer (ESE) method showed that the genetic variation might affect the efficacy of splicing factors (such as SRp 55 and SF2ASF1/2). As shown in Figure 5. The ASSP tool analysis showed that *XRCC3* rs861539 G>A may give rise to changes in the activity of putative splice sites. We found that score activation was 3.715 in the putative splice sites 102bp position of examined DNA sequence with rs861539 G allele, while the score activation changed to 3.272 in the same position with rs861539 A allele. The resulting change was that the putative splice site at 102 bp position changed from defined as unclasssified 3′ splice site to Alt.isoform/crytic 3′ splice site, which meaned rs861539 might have different post-transcriptional splicing regulation performance under different alleles. See Figure 6A,B.

**Figure 7:**
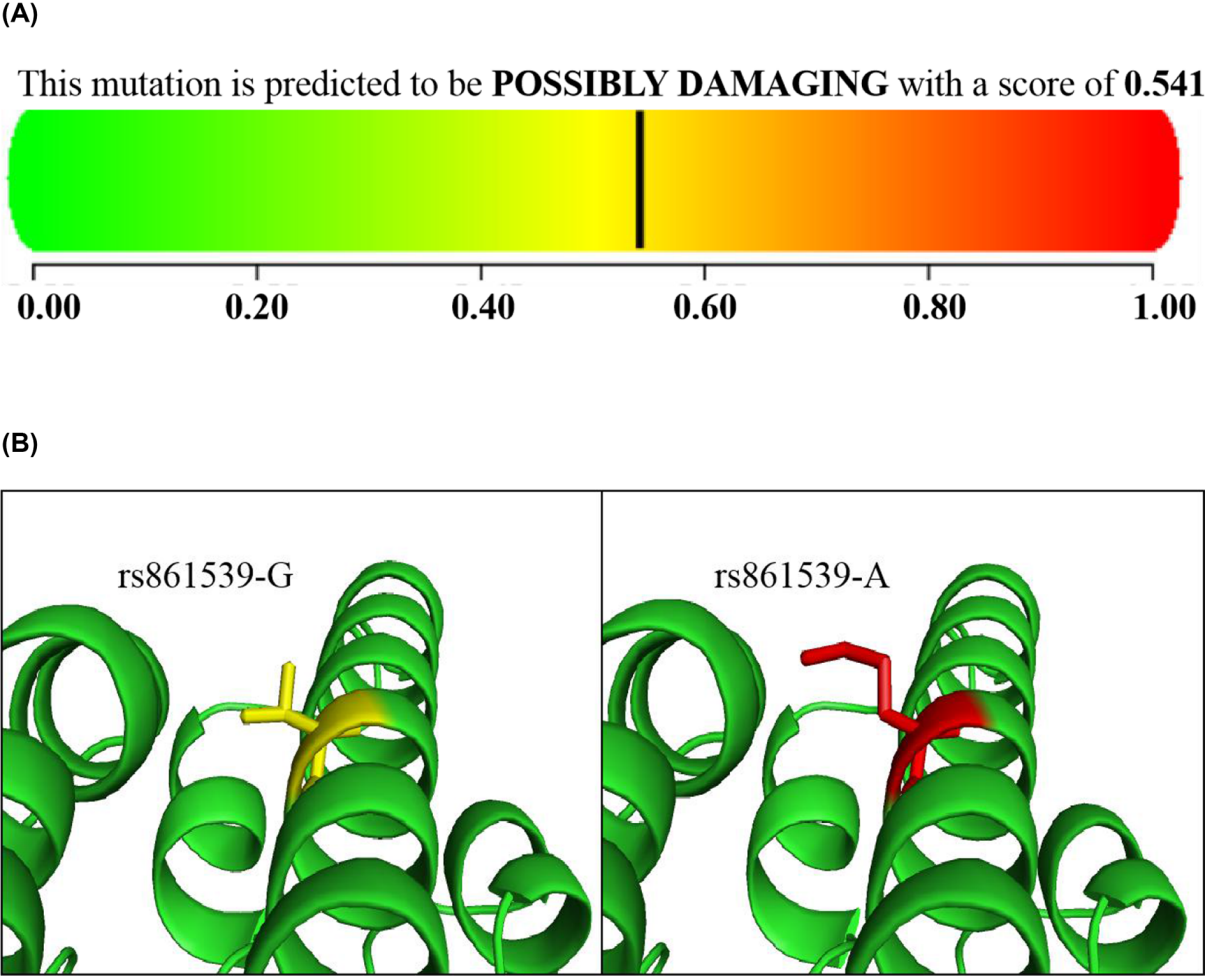
Analysis of the effect of rs861539 G>A on the structure of XRCC3 protein (**A**) Effect of Polyphen2 on the structure of XRCC3 protein. (**B**) Effect of Pymol on the structure of XRCC3.

Meanwhile, the GWAS4D prediction results revealed that rs861539 may act as an expression Quantitative Trait Loci (eQTL), affecting the expression of functional genes such as *XRCC3*, *MARK3*, *APOPT1*, *KLC1*, etc and was associated with the risk of OC in individuals (Table 4). In addition, the Polyphen2 tool analysis showed that the point mutation was possibly damaging (0.541) for structure and function of the XRCC3 protein. See Figure 7A. And the crystallographic structure changing from native amino acid to mutant amino acid analyzed by PyMOL Molecular Graphic -s System, Version 1.4.1 was shown in Figure 7B.

**Table 4:** The eQTL analysis on rs861539 and functional gene transcription levels based on GTEx v8 dataset

Position (rs861539)	Gene Name	Tissues or cells (*P*-value range: 10^−4^ to 1.694 × 10^−51^)
chr14:104165753	*XRCC3*	Muscle, Nerve, Artery, Adipose, Skin, Esophagus, Thyroid, Colon, Whole Blood, Lung, Prostate, Breast, Heart, Pituitary
chr14:104165753	*APOPT1*	Muscle, Cells (fibroblasts), Pituitary, Brain, Liver, Esophagus, Adrenal, Stomach, Spleen, Heart, Nerve
chr14:104165753	*BAG5*	Esophagus, Testis, Cells (fibroblasts)
chr14:104165753	*CKB*	Skin, Pancreas, Lung, Pituitary
chr14:104165753	*CTD-2134A5.4*	Thyroid
chr14:104165753	*KLC1*	Thyroid, Nerve, Spleen, Brain, Testis, Lung, Ovary
chr14:104165753	*LINC00637*	Colon, Thyroid
chr14:104165753	*MARK3*	Thyroid, Skin, Prostate, Pituitary, Nerve, Pancreas, Breast, Colon, Stomach, Lung, Adipose, Minor Salivary Gland, Ovary, Testis, Whole Blood, Brain, Spleen
chr14:104165753	*PPP1R13B*	Thyroid, Colon, Esophagus, Skin
chr14:104165753	*RP11*	Esophagus, Pancreas, Brain, Spleen, Lung, Heart, Cells (fibroblasts)
chr14:104165753	*TRMT61A*	Esophagus, Skin, Adipose, Nerve, Artery, Brain, Muscle, Whole Blood
chr14:104165753	*ZFYVE21*	Artery, Esophagus, Colon, Cells (fibroblasts), Muscle, Adipose

## Discussion

As a member of DNA damage repair genes family, the *XRCC3* plays a key role in maintaining the functional integrity of the human genome by participating in recombination repair after genomic DNA double-strand breaks damage [29]. It is believed that if the integrity of the *XRCC3* gene sequence or the function of the coding product is disturbed, its DNA damage repair efficiency will be weakened. Experimental study had also confirmed that *XRCC3*-deficient cells are more sensitive to cisplatin and chemoradiotherapy [30]. Studies showed that rs861539 variant of *XRCC3* gene was significant associated with the onset of multiple malignancies, such as gynecological malignancies, oesophageal cancer, prostate cancer, breast cancer, etc [5,7,31–33]. At present, there have been also a series of studies on the relationship between rs861539 and the incidence of OC, but the conclusions are contradictory.

In present meta-analysis, we confirmed that rs861539 was significantly associated with the risk of human OC under the most genetic models, particularly in Caucasians. The conclusion of the present study is similar to that of a previous meta-analysis [34] but is inconsistent with the meta-analysis conclusions of Yuan C [35] and Yan Y [36]; that is, there is no positive associations between rs861539 and OC risk observed. In comparison, most of the studies they included had relatively small sample sizes, while this study further increased the number of studies and the sample sizes. The larger sample size makes the statistical tests more efficient and the conclusions more reliable [37]. In addition, a sensitivity analysis was conducted in the meta-analysis, which showed that after excluding a certain research data, the pooled OR and its corresponding 95% CI did not fluctuate significantly. Moreover, we performed a FPRP as well as a TSA to make the findings more credible. It indicates that the conclusions that rs861539 significantly affects the risk of OC among women in this study are robust and reliable. Therefore, the early identification of risk genotypes or allele carriers of rs861539 accordingly is of great clinical and social significance for the early prevention and control of OC.

However, the biological mechanisms behind the significant associations between rs861539 and the susceptibility of women to OC, as well as the role of rs861539 in the pathogenesis of OC, have not yet been elucidated. In this study, we further utilized bioinformatics analysis to explore the possible biological functions of rs861539 and preliminarily revealed the potential molecular mechanisms by which rs861539 affects individuals' susceptibility to OC. According to the functional type and the gene region localization of rs861539, we explored its possible biological functions.

With the help of ‘SNP function prediction’ tool, it can be seen that rs861539 is located at the splicing site of *XRCC3* gene [8]. It was found that different alleles of rs861539 could lead to differences in the activity scores of *XRCC3* post-transcriptional related splicing sites and may produce a different splicing of the post-transcriptional mRNA sequence. This indicates that rs861539 has a significant regulatory effect on the post-transcriptional splicing of *XRCC3* gene, which may be one of the mechanisms to change the genetic susceptibility of individuals to OC. Meanwhile, eQTL analyses base on the GTEx database, which incorporates 127 tissue/cell type-specific epigenome data sets [27], suggests that rs861539 could regulate the expression of series of functional genes. In addition, as a missense mutation SNP, rs861539 G>A can change the encoded threonine (Thr) to methionine (Met). The analysis of PolyPhen2 and Pymol online tools showed that rs861539 variant brought about some changes in the structure of XRCC3 protein, which may cause damage to the DNA repair ability of XRCC3. Based on the above exploration, it can be revealed that rs861539 affects the DNA damage repair efficiency of XRCC3 by regulating the expression of *XRCC3* gene or changing the structure of the XRCC3 product. To sum up, rs861539 affects the post-transcriptional splicing of *XRCC3* gene and the expression of functional genes by changing the activity of splice sites and types of splicing factors. This evidence may provide new clues for revealing the mechanism of OC susceptibility.

This study still has some limitations. First of all, all studies included in this study have been published, and there may be some publication bias. Secondly, because the occurrence and development of OC is the result of multiple factors, such as genetic, environmental and endocrine factors, it is difficult to eliminate the effect of confounding factors in meta-analysis, which may affect the final results and conclusions. Also, evaluation of the potential gene–gene and gene–environment interaction effects on the risk of OC was limited because of lacking the original data of the reviewed studies. Finally, the present study only used bioinformatics methods to explore the mechanism of rs861539 affecting OC susceptibility, but did not carry out cell and molecular biological experiments. This weakened the strength of the evidence to some extent. Therefore, it must be cautious in accepting the conclusion of the present study.

## Conclusions

This study supports that *XRCC3* rs861539 G>A variant is significantly associated with the risk of human OC, especially in Caucasians. Involvement in the post-transcriptional regulation of *XRCC3* gene and the change of the structure of the encoded XRCC3 protein may be the biological mechanisms of rs861539 leading to individual susceptibility to OC. Our finds above provide new clues for researchers to further explore the biological mechanism of OC.

## Data Availability

The data used to support the findings of the present study are included within the article.

## Abbreviations

ASSP: Alternative Splice Site Predictor
CI: confidence interval
eQTL: expression Quantitative Trait Loci
ESE: Exonic Splicing Enhancer
NOS: Newcastle–Ottawa scale
OC: ovarian cancer
OR: odds ratio
PDB: Protein Data Bank
SNP: Single nucleotide polymorphism
XRCC3: X-ray cross-complementing group 3
